# Modulation of Gut Microbial Diversity through Non-Pharmaceutical Approaches to Treat Schizophrenia

**DOI:** 10.3390/ijms23052625

**Published:** 2022-02-27

**Authors:** Nayla Munawar, Aftab Ahmad, Munir Ahmad Anwar, Khalid Muhammad

**Affiliations:** 1 Department of Chemistry, College of Sciences, United Arabs Emirates University, Al-Ain 15551, United Arab Emirates; 2 Department of Biochemistry/Center for Advance Studies in Agriculture and Food Security (CAS-AFS), University of Agriculture, Faisalabad 38000, Pakistan; aftab.ahmad@uaf.edu.pk; 3 Industrial Biotechnology Division, National Institute for Biotechnology and Genetic Engineering, Constituent College of Pakistan Institute of Engineering and Applied Sciences, Faisalabad 38000, Pakistan; munir1@nibge.org; 4 Department of Biology, College of Sciences, United Arabs Emirates University, Al-Ain 15551, United Arab Emirates; k.muhammad@uaeu.ac.ae

**Keywords:** schizophrenia, gut microbiota, gut–brain axis, antipsychotics, neurotransmitters, psychobiotics, probiotics, prebiotics, polyphenols

## Abstract

Schizophrenia (SCZ) is a psychotic syndrome with well-defined signs and symptoms but indecisive causes and effective treatment. Unknown underpinning reasons and no cure of the disease profoundly elevate the risk of illness. Gut microbial dysbiosis related metabolic dysfunction is providing a new angle to look at the potential causes and treatment options for schizophrenia. Because of the number of side effects, including gut dysbiosis, of traditional antipsychotic drugs, new alternative therapeutic options are under consideration. We propose that non-pharmacotherapy using biotherapeutic products could be a potent treatment to improve cognitive impairment and other symptoms of schizophrenia. Use of live microorganisms (probiotics), fibers (prebiotics), and polyphenols alone or in a mixture can maintain gut microbial diversity and improve the two-way relationship of the gut microbiota and the central nervous system. Fiber and polyphenol induced management of gut microbiota may positively influence the gut–brain axis by increasing the level of brain-derived neurotrophic factors involved in schizophrenia. Furthermore, we endorse the need for comprehensive clinical assessment and follow-up of psychobiotic (pro and prebiotics) treatment in mental illness to estimate the level of target recovery and disability reduction in schizophrenia.

## 1. Introduction

Over recent years, the prevalence of neurological and psychotic disorders has increased worldwide. Among other brain disorders, schizophrenia (SCZ) is considered one of the most complex and difficult to treat phycological diseases [1]. It has been ranked in the top 10% of illnesses by World Health Organization (WHO) that affect 1% of the global population and the highest disabling and economically shattering health disorders [2]. Schizophrenia is characterized by distortions in thinking, disturbances of emotions, disorganized behavior, disorganized speech, and adverse hallucinations and delusions [3]. Most of the symptoms start developing in early adolescence to mid-30s and vary across individuals. If left untreated, they may worsen with time leading to suicidal thoughts. Generally, schizophrenia affects 60% of men and 40% of women. Men have an early onset of psychological signs and mostly show negative or cognitive symptoms leading to neurophysiological impairment. Whereas, females exhibit hallucinations and delusions (positive symptoms). The onset of the disease is normally observed between 18 and 25 years old in men and 25–35 years old in women, with a second peak close to menopause [4]. Men usually show an earlier onset of symptoms than women, but most of the time these symptoms are confused with other psychological disorders because of the prevalence of schizophrenia with other mental disorders such as anxiety, depression, obsessive compulsive disorder (OCD), and posttraumatic stress disorder (PTSD) [5]. Studies have suggested that the life expectancy of schizophrenic individuals is shortened by an average of 28 years [6]. Despite current scientific and technological evolution, the exact cause of schizophrenia is still unknown. Research indicates that this mental illness occurs due to a combination of physical, genetic, physiological, and environmental factors, thus is known as a multifactorial disease [7]. However, all multidirectional research on heritability, genetic variation, epigenetics, and the contribution of environmental factors to understand the etiology of the disease remains non-conclusive, provoking the need to explore new dimensions of research and explain the manifestations of schizophrenia. The lack of a basic understanding of the pathophysiology of the disorder hinders the curative treatment or prevention required for most people suffering from schizophrenia.

**Figure 1:**
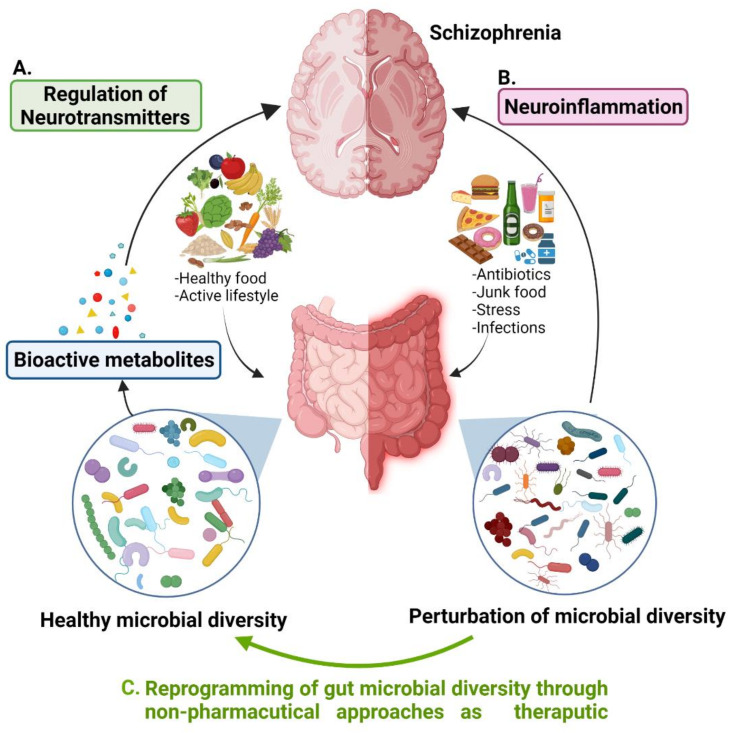
Association of gut microbial diversity to schizophrenia. (**A**) Healthy gut microbial species regulate neurotransmitters through bioactive metabolites produced by them. (**B**) Perturbation of gut microbial diversity causes neuroinflammation that might lead to schizophrenia. (**C**) Reprogramming of gut microbial diversity could be a potential therapeutic approach for the treatment or management of SCZ.

The mammalian gut microbiome has emerged as a key regulator of the host’s physiology. Trillions of microorganisms living in the human gut (gut microbiota) have constant communication between each other and their hosts that maintain a mutualistic symbiotic relationship among them [8]. It is believed that every individual has a characteristic commensal microbiota that has a unique composition depending on the food preferences, eating habits, lifestyle, and intestinal environment of that individual [9]. The presence of different types of microbial species generates a vast microbial diversity in the human gut that produces essential nutrients and unique bioactive metabolites. Microbial metabolites play a potent role in interspecies and microbial–host communication and subsequently have significant effects on the host’s health (Figure 1A). The commensal gut microbial diversity could be disrupted (dysbiosis) by infections, changes in eating habits and type of food, use of antibiotics, and stress that causes many diseases including psychotic disorders such as schizophrenia (Figure 1B). Mounting evidence suggests that perturbations of gut microbiota and their metabolites cause neuroinflammation and might contribute to the pathogenesis of SCZ [10]. A significant elevation or decreased level of some microbial species in the gut has exclusively been related to the risk and symptom severity of the disease [11,12] that confers the role of gut microbiota modulation in SCZ etiology. Now the question arises that if the fluctuation in gut microbial diversity is related to SCZ, would remission of this fluctuation provide an opportunity to reduce the risk or symptom severity of the disease? If so, what strategies should be adapted to reprogram the healthy gut microflora? It is highly plausible that the ability to alter the gut microbiome could lead to new therapeutic modalities for the prevention of chronic metabolic diseases and CNS-related disorders. The main focus of this article is to highlight the relationship between the alteration in gut microbial diversity, neurotransmitter dysregulation, and schizophrenia with the utmost emphasis on gut microbial diversity remodulation strategies by non-pharmaceutical approaches such as new therapeutics. We look at research studies that elucidate the significance of specific gut microbial species in SCZ through the regulation of neurotransmitters and how these two players interact with each other. Moreover, we explore the research that raised attention on antipsychotic-induced gut dysbiosis and metabolic dysfunction causing further health issues in SCZ patients that need to be fixed. Finally, we discussed potential gut microbial diversity reprogramming approaches for manipulating the relationship between the gastrointestinal (GI) tract and central nervous system (gut–brain axis) with probiotic, prebiotic, and polyphenol techniques, which represent alternative biotherapeutic possibilities for the treatment or management of SCZ (Figure 1C).

## 2. Methodology

For this chronicle review, the literature search protocol, and data analysis strategy was adapted according to Mohar et al. [13]. We selected articles published between 2018 and 2021 that referred to the relationship between schizophrenia and gut microbiota. The literature search of original research articles and review articles was performed using PubMed, Scopus, Google Scholar, and Web of Science to search for the latest publications of interest. To obtain comprehensive data according to the concept of this article, the search keywords used were gut–brain axis, neurotransmitters, antipsychotics, gut dysbiosis, non-pharmaceutical therapeutics, and schizophrenia. In total, 67 articles were selected to review originally. Because of the low number of original articles published in the above-mentioned period, addressing the question “how is the gut microbiome affected by antipsychotics and could it be reprogrammed by alternative therapeutics of pro/prebiotics and polyphenol supplements?”, a manual search was also performed, reviewing the reference lists of comprehensive review articles. Publications cited in the bibliographies of these articles and other psychotic and gut–brain axis papers were consulted when they addressed (a) the influence of gut microbiota on neurotransmitters, (b) the effect of antipsychotics on gut microbiota, (c) the composition of gut microbiota in schizophrenia, and (d) proposed treatments for gut dysbiosis. Original research articles were preferred and studies not conforming to the objectives of the review were excluded.

## 3. A Tridimensional Relationship of Gut Microbial Diversity, Neurotransmitters, and Schizophrenia

**Figure 2:**
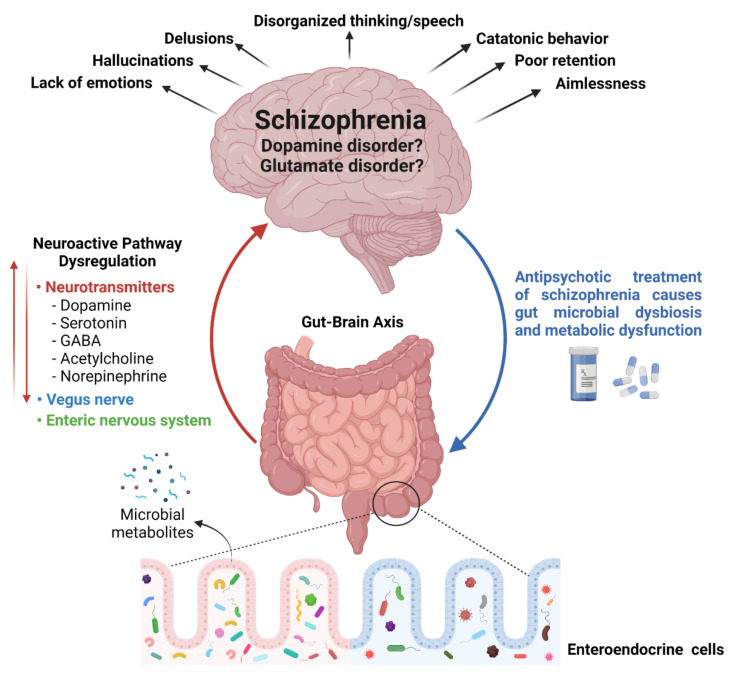
Relationship between gut microbial diversity and schizophrenia: A tridimensional association between gut microbial diversity, neurotransmitters, and the brain (left hand side). Antipsychotic-induced gut microbial dysbiosis (shown with blue arrow on right hand side).

In the late 19th century, schizophrenia was described as a disorder of thought and feelings that was understood by psychiatrists of that time as four ‘a’s—disturbances of associations, affect, ambivalence, and autistic isolation [14]. In the 20th century, after focusing on brain chemistry, schizophrenia was considered a “dopamine disorder”. Consequently, all neuroleptics, conventional and atypical, were yielded to block the dopamine D2 receptor. Although antipsychotics reduced delusions and hallucinations reliably, they failed to improve working memory and attention problems. With the focus on cognitive problems, schizophrenia was then hypothesized as “glutamate disorder”. It was proposed that the cognitive symptoms of the disease are a result of low activity of the N-methyl-D-aspartate (NMDA) receptor on gamma–aminobutyric acid (GABA) inhibitory interneurons in the prefrontal cortex. Unfortunately, the race of drug development for the disease hindered the research progress to determine the actual reason for the pathophysiology of the disorder. Therefore, a clear relation of dopamine D2 receptors or interneuron NMDA receptors to the root of this disorder remains to be elucidated. Post-mortem studies favor the “glutamate hypothesis” because of the constant observation of loss of GABA and reductions in key enzymes of glutamate biosynthesis in the samples. However, the changes observed in post-mortem studies might be the effects of chronic illness or consequences of treatment strategies rather than the cause of the disorder [15]. Various genetics, epigenetics, and environmental factors have been explored by several researchers worldwide to recognize the exact root of the disorder without success [16]. Since the number schizophrenic patients is increasing globally, it is of utmost importance to determine the exact reason for the illness to establish an effective treatment of the disease. During the search regarding the basis of schizophrenia, an interesting relationship between gut microorganisms and the central nervous system has been revealed, known as gut–brain axis. Bioactive metabolites produced by diverse microbial species residing in the human gut not only affect the host’s metabolism and immune system but also the central nervous system by various neurotransmitters [17] (Figure 2).

Alteration in glutamate metabolism has been related to modulation of the gut microbiota. For example, the lower levels of *Campylobacter jejuni* and *Bacteroides vulgatus* in the GI tract affects glutamate synthesis and its metabolism. Moreover, the conversion of L-glutamate into D-glutamate by glutamate racemase present in gut microorganisms such as *Brevibacterium lactofermentum*, *Bacillus subtilis*, *Corynebacterium glutamicum*, and *Brevibacterium avium*, also disturb glutamate metabolism and cause neuropsychiatric disorders [18]. The role of gut microorganisms in neurotransmitter regulation observed in different studies, that are described below, is summarized in Table 1. Gut–brain modules (GBMs) are neuroactive compounds that are produced or regulated by the gut microbiota having an influence on the central nervous system. The gut microbiome association with schizophrenia through gut–brain modules (GBMs) has been described by Zheng et al. [19]. The authors have observed a decreased microbiome α-diversity index and marked disturbances of gut microbial composition in 63 patients with SCZ versus 69 healthy controls (HCs). The use of Linear discriminant analysis Effect Size (LEfSe) to identify discriminating gut microbial species between SCZ and HCs identified 77 differential OTUs (operational taxonomic units) between the two groups. Twenty-three OTUs that mainly belonged to the bacterial taxonomic families Veillonellaceae, Prevotellaceae, Bacteroidaceae, and Coriobacteriaceae were increased in SCZ patients compared to HCs, however, 54 OTUs that belonged to the bacterial families Lachnospiraceae, Ruminococcaceae, Norank, and Enterobacteriaceae were decreased in SCZ patients compared to HCs. The presence of Veillonellaceae and Lachnospiraceae has exclusively been associated with SCZ severity. Very interestingly, the authors have observed an alteration in glutamate, glutamine, and GABA levels in the hippocampus when fecal matter from schizophrenic patients was transferred to germ-free mice suggesting that the alteration in SCZ microbiome alters neurochemistry and neurologic function through neurotransmitters. A metagenomic analysis of the fecal microbiome of 90 medication-free schizophrenia patients and 81 healthy controls conducted by Zhu et al. [20] identified 11 bacterial species, *Akkermansia muciniphila*, *Bacteroides plebeius*, *Veillonella parvula*, *Clostridium symbiosum*, *Eubacterium siraeum*, *Cronobacter sakazakii/turicensis*, *S. vestibularis*, *Alkaliphilus oremlandii*, *Enterococcus faecium*, *Bifidobacterium longum*, and *Bifidobacterium adolescentis*, significantly enriched in schizophrenia and some of these microbial species have been found significantly associated with symptom severity, cognitive performance, and diagnosis. *Streptococcus vestibularis* was exclusively identified as a schizophrenia-associated bacterium. Transplantation of *S. vestibularis* ATCC 49124 in the gut of C57BL/6 mice after antibiotics-based microbiota depletion, highlighted 11 GBMs involved in the abnormal behavior of mice through synthesis and degradation of several types of neurotransmitters related to glutamate synthesis, GABA degradation, and isovaleric acid synthesis, in the same study [20]. Notably, a significant difference in the gut microbiome of metabotropic glutamate receptor 5 (mGlu5) knockout (KO) schizophrenia model mice and wild type (WT) has been identified. A decreased relative abundance of the *Erysipelotrichaceae* family and *Allobaculum* genus in a mouse model of SZ was identified through 16S rRNA sequencing of bacterial genomic DNA from fecal samples of KO and wild type mice. The signature bacteria that discriminated between both groups consist of the *Erysipelotrichales*, *Bacteroidales*, and *Clostridiales* orders. Metabotropic glutamate receptors (mGluRs) exert fine control over glutamate activity. Gut dysbiosis in KO mice indicates a link between glutamate regulation and gut microbiota that was disturbed in the mGlu5 KO mouse, thus resulting in gut dysbiosis [21].

Including emotions, learning, and memory, several brain functions are controlled by neurotransmitters [17,22]. Gamma-aminobutyric acid (GABA), dopamine, glutamate, acetylcholine, norepinephrine, and serotonin are the most common gut microbe regulated neurotransmitters that influence cognition [23]. Serotonin (5-HT) dysfunction is known to be involved in the development of schizophrenia and antipsychotics such as olanzapine and risperidone target 5-HT2A and 5-HT1A receptors to ameliorate negative symptoms and mood disturbances in schizophrenia. A 5-HT2A antagonistic drug enhanced the release of dopamine in the striatum by decreasing the inhibitory effect of serotonin [24], indicating the potential role of serotonin and dopamine in schizophrenia pathogenesis. An increased level of tryptophan, a precursor of serotonin, was observed in *Biofidobacterium infantis* treated rats compared to controls showing an association of gut microbiome, neuroactive compounds, and the central nervous system [25,26]. Moreover, *Lactobacillus* and *Bifidobacterium* species in the gut are known to regulate GABA [27], *Bacillus* and *Serratia* control dopamine [28], norepinephrine is regulated by *Saccharomyces*, *Escherichia coli*, and *Bacillus*, and serotonin production is related to *Candida*, *Escherichia*, *Streptococcus*, and *Enterococcus* species in the gut [29]. Lower levels of tryptophan and serotonin in serum and elevated levels of dopamine and serotonin in the prefrontal cortex and hippocampus, respectively, was observed in mice transplanted with fecal microbiota from patients with schizophrenia that also displayed impaired learning and memory abilities in the treated mice [20]. Gut microbiota affects the synthesis and secretion of serum serotonin [25,30,31,32] and influences the serotonergic system by altering tryptophan availability in the plasma [33]. The induction of schizophrenia symptoms with NMDA receptors inhibition indicates a link between hypofunction of NMDA receptors and schizophrenia. This connotation is known as the “NMDAR hypofunction hypothesis” [34]. Numerous findings indicate that SCZ is induced by NMDAR hypofunction using drug treatments [35,36,37,38,39]. Indeed, hypoactivity of NMDA and brain-derived neurotrophic factor (BDNF)/glial-cell derived neurotrophic factor (GDNF) receptors via gut microbiota alterations in central nervous system disorders including schizophrenia has also been reported [40,41,42].

Collecting all evidence available so far, in our opinion, schizophrenia is not caused by the aberrant function of a single neurotransmitter. To elucidate the origins of SCZ-cognitive inabilities, investigations based on one-line neurotransmission are likely an outdated approach. A complex experimental design covering different neuroactive players is required to uncover the pathology of this wide-spectrum symptomatology containing disease. It is evident that the gut microbiota coordinates with the central nervous system (CNS) through the vagus nerve [43] and the enteric nervous system [44] by regulating neurotransmitter synthesis that is required for normal neuron plasticity and brain functioning (Figure 2). These neurotransmitters are capable of stimulating enteric cells that can modulate communication between ENS and CNS. However, the research indicating the exact microbial strains, that particularly regulate these neurotransmitters, is lagging behind. Large scale “omics” studies could facilitate in unveiling the process, but the unavailability of specific reference databases and tools to study microbiome neurotransmitter potential may hinder the precise interpretation of the data.

**Table 1:** A brief summary of gut microbial species that potentially affect the gut–brain axis through regulation of different neurotransmitters.

Gut Microorganisms	Role Associated with Neurotransmitter Regulation	References
*Campylobacter jejuni*	Affect glutamate synthesis and its metabolism	[15]
*Brevibacterium lactofermentum*, *Bacillus subtilis*, *Corynebacterium glutamicum*, and *Brevibacterium avium*	Involved in conversion of L-glutamate into D-glutamate through glutamate racemase enzyme, thus disturbs glutamate metabolism	[15]
*Streptococcus vestibularis*	Involved in synthesis and degradation of several types of neurotransmitters related to glutamate synthesis, GABA degradation, and isovaleric acid synthesis	[18]
*Erysipelotrichales*, *Bacteroidales*, and *Clostridiales*	Affect glutamate activity through metabotropic glutamate receptors (mGluRs)	[19]
*Lactobacillus* and *Bifidobacterium* species	Regulate GABA	[25]
*Bacillus* and *Serratia*	Control dopamine	[26]
*Saccharomyces*, *Escherichia coli*, and *Bacillus*	Regulate norepinephrine	[27]
*Candida*, *Escherichia*, *Streptococcus*, and *Enterococcus* species	Regulate serotonin production	[27]

## 4. Antipsychotics-Induced Side Effects and Gut Microbiota Dysbiosis in Schizophrenia

Because of the poor understanding of the etiology of schizophrenia, its treatment is restricted to the use of antipsychotic drugs and tranquillizers (neuroleptics or anti-schizophrenic drugs). Most widely used pharmacological treatments have not provided any evidence of substantial improvement and recovery in most people with chronic schizophrenia [23]. Instead, the use of antipsychotics has several side effects such as weight gain [45], diabetes mellitus [46], atherosclerosis [47], glucose metabolism dysregulation [48], and most importantly gut microbial dysbiosis [49]. The use of antibiotics and changing dietary habits are the main reasons for alteration in the gut microflora that is directly associated with modified levels of neurotransmitters and brain function [50,51,52] (Figure 2). The existing evidence shows that antipsychotic-induced gut microbial alterations initiate adverse metabolic events that have an impact on the psyche of the patients [53]. The correlation between altered gut microbiome and psychiatry is an emerging field of schizophrenia research. The significant potential of psychotic drugs to alter gut microbial composition in vitro has been observed by Macedo et al. [54] and Maier et al. [55]. An altered composition of gut microbiota in schizophrenia patients has also been observed very recently by Manchia et al. [56]. The composition of gut microbiota in 38 schizophrenia patients, 20 health controls, 18 patients with treatment-resistant schizophrenia (TRS), and 20 responders (R) to different antipsychotics has been investigated in detail. In line with previous studies, the presence of a distinct composition of gut microbiota in SCZ patients versus healthy controls, with several bacteria at different taxonomic levels only present in either one group or the other, has been reported by the authors. For example, bacteria of the genus *Haemophilus* were deficient in SCZ patients compared to the controls in this study, which is consistent with another study conducted by Nguyen et al. [57] where the gut microbiota of patients with chronic schizophrenia was compared to healthy controls. An increased level of *Papillibacter cinnamivorans* and *Erysipelatoclostridium ramosum* species was observed in treatment-resistant SCZ patients compared to healthy controls. Moreover, enrichment of the genus *Actinomyces* and *Porphyromonas* in the gut microbiota of TRS patients compared to those responsive to antipsychotics indicates a complex relationship of gut microbial diversity (GMD) related to schizophrenia, the pattern of treatment response, and the use of a specific class of antipsychotics in the disease. The influence of antipsychotics on gut microbiota in schizophrenia patients has been reported by Zhu et al. [58]. The author has identified 26 different microbial species in medication-free SCZ patients and healthy controls as baseline. After 3 months of antipsychotic treatment, 20 microbial species remained altered in patients compared to controls. Notably, 28 differentially abundant bacterial species were observed throughout the antipsychotic treatment, five of which were included in the 26 operational taxonomic unit SCZ classifiers. This observation led the authors to conclude that antipsychotics influence the gut microbiota but cannot restore it from SCZ-associated alterations [58]. Among several other researchers, the negative effects of antipsychotics on microbiota levels have also been observed by Ma et al. [59] through 16S rRNA gene sequence analysis of fecal samples from 40 first-episode drug-naïve SCZ (FSCZ) patients, 85 chronically antipsychotic-treated SCZ (TSCZ) patients, and 69 healthy controls (HCs). The increased abundance in the families *Enterococcaceae* and *Lactobacillaceae* and in the genera *Enterococcus*, *Escherichia*, *Lactobacillus*, *Shigella*, *Streptococcus*, and *Veillonella* in chronically antipsychotic-treated SCZ patients compared to first episode SCZ patients and healthy controls indicates the alteration in gut microbiome as a result of antipsychotics treatment.

Psychiatric patients are at higher risk of many metabolic syndromes, especially antipsychotic-induced weight gain (AIWG) that is linked to the use of antipsychotics such as olanzapine and clozapine [60,61]. Although the exact mechanisms of AIWG remain unclear, its link with antipsychotics-induced gut microbiota modulation has been illuminated in several studies. For example, a lower *Bacteroidetes*:*Firmicutes* ratio and an increase in BMI after chronic treatment of risperidone in male children (*n* = 18) has been reported by Bahr et al. [62]. Moreover, a significant increase in the relative abundance of fecal *Bifidobacterium* spp. and *Escherichia coli* and a significant decrease in the abundance of fecal *Clostridium coccoides* group and *Lactobacillus* spp. have been reported in first episode SCZ patients after a 24 weeks treatment with risperidone. Weight gain after treatment with risperidone was observed in patients that was exclusively correlated with the relative abundance of *Bifidobacterium* spp. in their gut [63]. On the other hand, the effect of olanzapine on the gut microbiome in 20 SCZ patients observed by Pelka-Wysiecka et al. [64] showed no significant changes in alpha diversity and GMB composition of the patients after 6 weeks of treatment, but a significant increase in BMI in women patients has been observed. The patients were washed out for 7 days, meaning taking no psychiatric medication and consuming a normal hospital diet. The stool samples after the wash-out period were taken as baseline for analysis and this data was compared with stool samples taken after 6 weeks of treatment with olanzapine. No significant change in the gut microbiota during this study has been related to the short intervention period of olanzapine treatment [65].

In addition to the few examples mentioned above, the current data available so far about microbial diversity in schizophrenia and antipsychotics-induced dysbiosis is complex, non-conclusive and conflicting, but provides strong grounds for further careful investigation to explore this new area of research. The inconsistency of results in different studies could be because of the use of different antipsychotics in each study that might have different effects on gut microbial diversity. Moreover, the effect of gut microbiota on the pharmacokinetics of antipsychotic medications should also be considered to draw an exact picture of the GMD–antipsychotic relationship. Cussotto et al. [66] noticed significantly increased bioavailability of olanzapine in rats that had antibiotic-induced depletion of gut microbiota. Nevertheless, a high level of olanzapine has been linked to the elevated risk of developing peripheral oedema, postural hypotension, and diabetes, thus it is warranted to consider its use in clinical practice [67]. The regulation of bioavailability of olanzapine by gut microorganisms indicates their significant role in drug metabolism to protect the host from drug-induced side effects. Therefore, robust evidence has been found regarding antipsychotic-induced altered composition of gut microbiota being associated with schizophrenia-related metabolic dysfunctions. Therefore, restoration of the gut microbiota can play a significant role in treating the symptoms and managing the antipsychotic-induced side effects in schizophrenia.

## 5. Gut Microbial Diversity Management Strategies to Treat Schizophrenia

The use of non-pharmaceutical molecules is trending once again because of their proven efficiency in disease management with reduced side effects and cost-effective capacity. The manipulation of gut microbiota with probiotics, prebiotics, and polyphenolic supplements could be a promising therapeutic option to manipulate gut microbial diversity and minimize psychotic symptoms and/or antipsychotic-induced side effects in schizophrenia patients. However, biotherapeutics bring their own challenges that need to be addressed before large-scale human trials. This section explores the potential of using live therapeutics (probiotics) and natural molecules (prebiotics and polyphenols) as nonpharmacological treatment options for schizophrenia.

### 5.1. Probiotics: Impact of Live Biotherapeutics on Disease Symptoms

**Figure 3:**
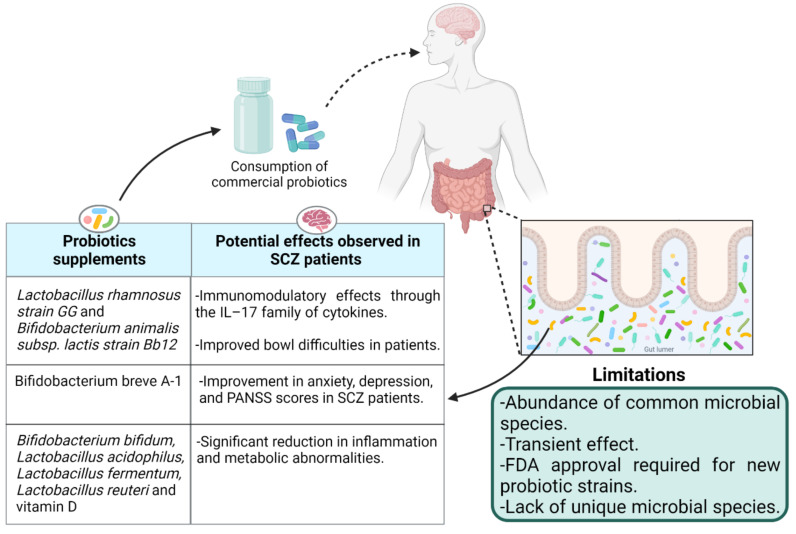
The effects of probiotic supplementation in schizophrenia patients and the limitations.

The use of probiotics to fix altered gut microbiota has gained enormous attraction after determining its role in health and disease. According to the World Health Organization (WHO), probiotics are live bacteria that provide health benefits to the host when administrated in an adequate quantity. The use of living bacteria to treat gut diseases is as old as World War I when Alfred Nissle, a German physician and scientist, used an *E. coli* strain to treat diarrhea [68]. Now, different gut-associated microbial strains, typically from the genera *Lactobacilli* and *Bifidobacteria*, are available commercially as supplements to manipulate the gut microbiota to treat gastrointestinal related diseases. The effect of probiotics in psychotic disorders has been investigated in both animal and human studies. The literature regarding the exclusive use of probiotics for the treatment of schizophrenia is limited thus far. The effect of probiotic supplementation in chronic schizophrenia patients was observed by Tomasik et al. [69]. A 14-week adjuvant treatment of 58 participants with probiotics (*Lactobacillus rhamnosus* strain GG and *Bifidobacterium animalis* subsp. lactis strain Bb12; *n* = 31) or placebo (*n* = 27) with their usual antipsychotic treatment identified immunomodulatory effects of probiotic supplementation in schizophrenia through the IL–17 family of cytokines. Probiotic supplements showed improved bowel difficulties in the patients, but no change in positive and negative syndrome scale (PANSS) psychiatric symptom scores was observed during the trial [69]. Similarly, no significant difference in PANSS scores but greater effects on positive symptoms than negative symptoms was reported by Severance et al. [70] when 56 patients with at least moderately severe psychotic symptoms were treated with a mixture of *L. rhamnosus* strain GG and *B. animalis* subsp. *lactis* strain Bb12 for 14 weeks. Interestingly, no significant difference in PANSS score in 58 patients having at least moderately severe psychotic symptoms (aged 18–65 years) by using the same probiotics supplements for 14 weeks (*L. rhamnosus* strain GG and *B. animalis* subsp. *lactis* strain Bb12) was observed in the past by Dickerson et al. [71]. However, another report by Okubo et al. [72] showed an improvement in anxiety, depression, and PANSS scores by treating SCZ patients with probiotic *Bifidobacterium breve* A-1 for 4 weeks. The authors related this improvement to IL-22 and tumor necrosis factor-related activation induced cytokines (TRANCE) that play a critical role in the function of the gut epithelial barrier. The improvement in total PANSS score and antioxidant activity in plasma has also been reported by Ghaderi et al. [73] when schizophrenia patients were treated with a mixture of probiotics *Bifidobacterium bifidum*, *Lactobacillus acidophilus*, *Lactobacillus fermentum*, and *Lactobacillus reuteri* and vitamin D supplements for 12 weeks. The authors reported a significant reduction in inflammation and metabolic abnormalities. However, it is unclear if probiotics or vitamin D alone or their combination is exactly responsible for the observed improvement in the patients (Figure 3).

The improvement of symptoms in some studies by using different microbial strains as probiotics compared to others with no improvement using the same type of microbial strains is indicating the strain-specific effects of probiotic supplements. The strain-specific immunomodulatory effects of probiotics are already documented [74]. Therefore, it is highly recommended to investigate the effect of every potential probiotic strain in schizophrenia and their ability to reduce symptoms of the disease. Very recently, Liu et al. [65] have reviewed the potential benefits of using psychobiotics for the treatment of schizophrenia. The author has documented multiple studies that focus on taxonomic groups that are significantly increased/decreased in schizophrenia. Notably, almost all studies show inconsistent results. For example, Ma et al. [59] reported the increase in microorganisms of the family *Enterococcaceae* in schizophrenia, but Xu et al. [75] reported the decreased level of this family in schizophrenia. However, lower levels of the family *Lachnospiraceae* in SCZ patients have been reported in three different studies [18,76,77] that have been reviewed by Ng et al. [78]. The *Lachnospiraceae* family is known for its health benefits by butyrate and other short-chain fatty acid (SCFs) production. Moreover, *Roseburia* (*Lachnospiraceae*) has been reported to contribute to the integrity of the intestinal barrier, indicating that the depletion of *Lachnospiraceae* in schizophrenia has a potential role in the pathogenesis of the disease. Use of probiotic supplements appears effective and safe to reduce antipsychotic-induced metabolic disturbances. Schizophrenia patients with a drug-naïve first episode were randomized and treated with olanzapine (15–20 mg/day at 8:00 p.m.) and olanzapine plus probiotics capsules containing *Lactobacillus*, *Bifidobacterium*, and *Enterococcus* at least 5.0 × 10^7^ CFU/g, for 12 weeks [79]. The authors did not find any efficacy of probiotic supplements in weight gain of the patients, however, the increase in mean fasting insulin was significantly lower in patients treated with antipsychotic plus probiotics compared to patients having olanzapine monotherapy, showing the effectiveness of probiotics in attenuating antipsychotic-induced elevation of fasting insulin and insulin resistance [79].

It is evident that *Lactobacillus* and *Bifdobacterium* species are capable of producing neurotransmitters such as gamma–aminobutyric acid (GABA) and acetylcholine, which directly target receptors in the central nervous system [26]. Because of these obvious benefits, most of the researchers used *Lactobacillus* and *Bifdobacterium* species in different combinations to investigate the effect of probiotic supplements in schizophrenia, ignoring the exact depleted strains in the gut during disease. One possibility of this naivety could be the unavailability of depleted strains as probiotic supplements. Identification and cultivation of new gut microbial species was expanded in recent years due to the probiotic’s potential. However, the requirement of rich growth media, anaerobic conditions for growth, and optimization of the fermentation process at a large manufacturing scale is complex and costly, thus not favorable to industrialists. Moreover, long-term intervention of probiotics is required because of their transient effect [80] that limits the use of probiotic supplements for commensal microbiota management. Additionally, obtaining permission from the Food and Drug Administration (FDA) and the European Directorate for the Quality of Medicines to introduce any novel probiotic candidate that has not been used before is another challenge that hinders the availability of novel probiotic strains as biotherapeutic products (Figure 3). Therefore, the use of prebiotics to maintain a healthy commensal gut microbiota is endorsed to avoid gut-related disorders.

### 5.2. Prebiotics: A Connection between Dietary Fiber, Gut Microbiota, and Schizophrenia

**Figure 4:**
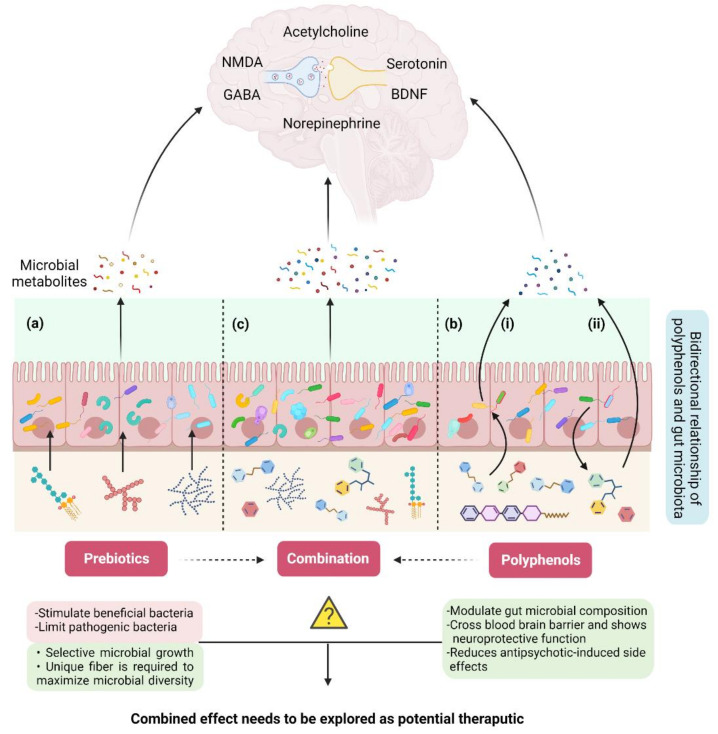
Remodulation of the gut–brain axis through non-pharmaceutical approaches. (**a**) Intake of prebiotics (non-digestible fiber) modulate gut microbial diversity at a limited scale. (**b**) Bi-directional relationship of polyphenols and gut microbiota: i. polyphenols affect the growth and metabolism of gut microbiota and modulate their composition and function, ii. gut microbiota metabolizes specific polyphenols and converts them into bioactive compounds with health benefits. (**c**) Use of prebiotics and polyphenols together could be a powerful strategy for in situ reprogramming of gut microbiota to treat SCZ.

The profound impact of dietary fiber on human health through the gut–brain axis has been well documented [81,82,83,84]. Non-digestible soluble or insoluble carbohydrates that are selectively utilized by gut microorganisms conferring a health benefit by influencing the entire metabolism of the host are known as prebiotics [85]. The Food and Drug Administration (FDA) authority defines prebiotics as non-digestible dietary fiber that could be soluble or insoluble plant carbohydrates and lignin or isolated or synthetic non-digestible carbohydrates that have beneficial physiological effects for human health [86]. Prebiotics are oligo/polymers of carbohydrates that could be obtained from food and are resistant to enzymatic activity in the human upper gastrointestinal tract, thus neither digested nor absorbed by the small intestine. Consequently, they end up in the colon, fermented by the intestinal microflora, and positively regulate the composition and activity of the specific intestinal microbiome that cause beneficial interactions with the host through the production of short chain fatty acids and vitamins [87] (Figure 4a). Prebiotics also have an impact on human health by stimulating beneficial bacteria and limiting pathogenic bacteria, thus playing a vital role in bacterial homeostasis by sustaining a healthful microbiome [88,89]. On the basis of chemical composition, constituent units, origin, and glycosidic bonds, the prebiotics are classified into three categories such as oligosaccharides (fructooligosaccharides (FOS), galactooligosaccharides (GOS), xylooligosaccharides (XOS) etc.), fibers (pectin, dextrin, etc.), and polyols (mannitol etc.) [89,90]. The dietary fibers that are involved in the enhanced production of SCFAs through the gut microbiota are considered a more potent type of prebiotic for health benefits and in disease control. Many lines of evidence show the involvement of the immune system and inflammation in the pathophysiology of schizophrenia that could be targeted from the treatment point of view. For example, a very high level of circulating C-reactive protein (CRP) in schizophrenia patients has been observed. CRP is considered a reliable biomarker for subclinical systemic inflammation as it identifies inflammatory stimuli and protects the host as a part of the innate immune system [91]. In contrast to the use of antipsychotics [92], the elevated level of CRP in various human diseases has been decreased by using prebiotics [93], showing their potential as an alternative anti-inflammatory therapeutic agent to attenuate symptoms that cannot be controlled by medication in schizophrenia. Neuropsychological effects and assistance in the management of cognition and weight gain in schizophrenia by prebiotics have been reviewed by Kao et al. [94,95]. Improved cognitive flexibility and increased cortical NMDA receptor functioning in rats have been reported by [96] after three weeks of daily prebiotic administration. Recent studies have shown that increased intake of dietary fiber improved cognitive and mental health quality in patients with mental, physical, and gastrointestinal disorders [97,98,99].

Interestingly, the specific sources of dietary fibers seem to be differentially associated with the modulation of depression [98], strongly indicating a correlation between the specific type of fibers and selective growth of specific beneficial microbial species or strains in the gut. Very recently, La Torre et al. [100] reviewed dietary fiber studies on cognitive and affective processes. Despite the variation in the intervention period and types of fiber supplements used, the majority of these studies showed positive effects on affective and cognitive indices, highlighting the beneficial effects of fiber on mood and cognition. However, the potential mechanisms of action of fibers in most studies have not been discussed. Most likely, dietary fiber stimulates affective and cognitive processes through its interaction with the gut microbiome [100]. The gut microbial modulation effect of inulin (fructan) on mice with SCZ has been studied by Guo et al. [101]. The authors have demonstrated that inulin alleviated the damage of brain neurons after 6 weeks of treatment of SCZ mice with inulin. An improved intestinal integrity and permeability, anti-inflammatory effects with decreased levels of TNF-α, IL-1β, and IL-6 in plasma and brain tissues, and enhanced levels of neurotransmitters in the inulin-treated group compared to the control has been related to the improvement in psychotic symptoms and impaired learning in this study. The authors also spotted that dietary inulin restores the gut dysbiosis by increasing *Bifidobacterium* and *Lactobacillus* and decreasing *Akarnania* and *Eubacterium-fissicatena*. The increased abundance of *Akarnania*, a mucin-degrading bacterium, has been exclusively related to the pathological process of schizophrenia in this study that was managed after inulin intervention. Additionally, a decrease in body weight in inulin fed mice compared to the control group could be associated with an increase in *Bifidobacterium* and *Lactobacillus* bacteria in inulin treated mice. Normally, prebiotic fibers are metabolized by *Lactobacillus* and *Bifidobacterium* in the intestine, therefore, their abundance increased in the gut after fiber intervention. Increased abundance of both microbial species has already been associated with better memory and lower anxiety in rodents [102] and humans [103]. Bacterial strains exert positive effects on memory and anxiety through their metabolites such as SCFAs and brain-derived neurotrophic factor (BDNF) that arbitrate the interaction between the gut microbiota and the central nervous system.

Specific microbial strains produce specific postbiotics (metabolites) having particular psychological functions. As it has been mentioned above that the elimination or decreased abundance of some specific microbial species is attributed to schizophrenia and its related problems such as constipation and weight gain, it is essential to improve the growth of particular strains and enhance healthy gut microflora naturally. In our previous article, we have mentioned the urge of investigation of unique prebiotic compounds that can maximize gut microbial diversity and stabilize the growth of rare microbiota related to schizophrenia [15]. Moreover, an improved understanding of the relation between prebiotics and probiotics is required for the selection of better oligosaccharides with varied functional properties as therapeutic agents to manage CNS disorders such as schizophrenia. A great health impact has encouraged high enthusiasm in prebiotics research and innovation that will boost the prebiotics market in the near future. Scientists are adapting various methods to produce novel prebiotic supplements that could enrich the microbial diversity [104]. However, consumption of fiber-rich whole food not only offers a variety of fibers but also minerals and antioxidants that have a great influence on mood and cognition [105].

### 5.3. Polyphenols: A Potential Interplay between Polyphenols, Gut Microbiota, and Schizophrenia

In addition to fiber, gut microorganisms metabolize other naturally occurring compounds such as polyphenols that are the most abundant antioxidants present in human food. Polyphenols are a heterogenous group of organic molecules containing multiple phenol structural units. More than 8000 polyphenol compounds have been identified so far that have been classified into different groups based on their carbon skeleton and structural complexity [106]. Bioavailability of polyphenols is linked to their structural complexity and most of the polyphenols are not absorbed in their native form. Although smaller polyphenols can be absorbed in the small intestine, complex polyphenols reach the colon without any modification, where the gut microbiota metabolize them to produce low-molecular-weight, bioactive and bioavailable metabolites containing health benefits [107]. Gut microbiota and polyphenols have a bidirectional relationship as gut microbiota metabolize complex polyphenols making them bioavailable to produce health benefits, while polyphenols modulate gut microbial composition and functions influencing their growth, metabolism, and restricting the growth of pathogens [108] (Figure 4b).

Along with several other activities such as antimicrobial, anti-inflammatory, antioxidant, and antidiabetic, the neuroprotective potentials of dietary polyphenols have also been documented in the last couple of years and reviewed recently [109,110,111]. Polyphenols play an important role in memory, learning, and cognitive functions by protecting neurons against injury and inflammation [112]. Many studies highlighted the beneficial role of polyphenols in oxidative stress signaling and expression of antioxidant enzymes, thus ultimately leading to maintenance of brain homeostasis [113]. It has also been reported that polyphenol metabolites carry neuroprotective functions by crossing the blood–brain barrier [114]. As polyphenols play an important role in neuron protection, there are growing interests in understanding the relationship between dietary polyphenols, gut microbiota, and their importance in mental disorders. Quercetin, a plant flavonoid, has been shown to increase gut microbial diversity and relative abundance of *Glutamicibacter*, *Facklamia*, and *Aerocorrus*, increase hippocampal BDNF, and improve learning and memory [115]. Thus far, there is no direct evidence that an interruption in dietary polyphenols supply is associated with the pathophysiology and patho-biochemistry of schizophrenia in human beings. However, preclinical studies demonstrate the protective role of polyphenols in antipsychotic-drug-induced side effects in schizophrenia patients. Loftis et al. [116] established the usefulness of epigallocatechin gallate (EGCG), a green tea extract, as an adjunct to antipsychotic medication maintenance in schizophrenia patients. The authors observed a significant reduction in psychotic, depressive, and anxiety symptoms in both EGCG and placebo groups that was associated with reduced expression of cytokines. During this study, adults with schizophrenia, schizoaffective disorder, or bipolar disorder, who were maintained on antipsychotic and other psychotropic medications, were randomized to supplemental EGCG or placebo. In this trial, 34 participants (17 EGCG, 17 placebo) were randomized, and 25 participants (14 EGCG, 11 placebo) completed the study. Patients were given 150 mg EGCG in a capsule containing some microcrystalline cellulose (MCC) and placebo capsules having the same amount of MCC, for 8 weeks. No significance difference in treatment was observed in both EGCG and placebo groups, indicating a lack of antipsychotic or other psychotropic properties of EGCG. However, no observable difference in treatment was attributed to the small sample size of the study by the authors. Since psychotic symptoms were improved in both groups, the role of microcrystalline cellulose (MCC) given to both groups should also be investigated in our opinion. MCC is known to provide positive effects on gastrointestinal physiology by influencing the expression of enzymes involved in lipid metabolism [117]. The improvement in psychotic symptoms and no observable difference in treatment during this study may be indicating the same mechanism of action adopted by EGCG and MCC that led to the improvement of psychotic symptoms in all participants. This proposition not only highlights the significance of MCC in the maintenance of psychotic symptoms in schizophrenia that needs to be studied further but also indicates the requirement of a careful selection of control supplements to avoid any biased observations in human trials in the future.

Since polyphenols have antioxidant activity, they could be potent candidates to explore their potential for managing antipsychotics-induced oxidative stress disorders. Only a few studies investigated the role of polyphenols in side effects associated with long-term use of neuroleptics (antipsychotic medications). For example, Tardive dyskinesia (TD), uncontrollable stiff, jerky movements of the face and body, is associated with long-term use of neuroleptics. Although the major cause of antipsychotic treatment-induced TD is not clear, however, free radicals are reportedly involved in the pathophysiology of TD. Bishnoi et al. [118] confirmed that oxidative stress is involved in the development of haloperidol-induced orofacial TD. The results showed that chronic administration of haloperidol induced oxidative damage in the rat brain, which can be inhibited by curcumin. The authors proposed curcumin as a possible therapeutic treatment of haloperidol’s induced side effects. Similarly, a flavonoid, quercetin (3,5,7,3,4, pentahydroxy flavone), inhibited haloperidol-induced catalepsy associated with schizophrenia [119]. In addition, in ex vivo experiments, haloperidol-induced lipid peroxidation in human plasma was reduced by quercetin and resveratrol (3,4,5, trihydroxy stilbene). A flavonoid, epicatechin, from green tea also inhibited haloperidol-induced lipid peroxidation in human plasma in ex vivo [120]. Polyphenols from berries also reversed ziprasidone (an antipsychotic drug)-induced lipid peroxidation in human blood in ex vivo experiments [121]. Although these studies show the potential of polyphenols to reverse drug-induced side effects during schizophrenia treatment, however, results from ex vivo are not a true picture of in vivo conditions because during in vivo, polyphenols are metabolized into their derivatives while passing through the GI tract. Chen et al. [122] demonstrated that the extract from Gingko biloba (EGb-761), can cross the blood–brain barrier and protect brain from damage from oxidative stress. Moreover, the ginkgo biloba extract, EGb761, increases synaptosomal uptake of 5-hydroxytryptamine in both in vitro and ex vivo studies [123] indicating that EGb-761 can have a positive effect on psychotic symptoms through the tryptophan pathway. All these studies demonstrate the potential of polyphenols in reducing the side effects of antipsychotic drugs and protecting the brain from oxidative stress.

The modulation of gut microbiota by polyphenols has been reviewed very recently by Wan et al. [124]. The ability of polyphenols as a nonviable food component to modulate gut microbiota and confer health benefits, categorizes them as “prebiotics”. In several studies, it has been observed that the intervention of polyphenols reduces pathogenic microbial species in the gut without affecting beneficial bacteria or could increase the number of commensal microbiota [125,126,127,128,129]. Queipo-Ortuno et al. [130] reported the lower level of *Firmicutes*, intestinal microorganisms associated with metabolic disorders, with the ingestion of grape seeds and wine polyphenols. While, a significant increase in *Enterococcus*, *Prevotella*, *Bacteroides*, *Bifidobacterium* spp., and *Blautia coccoides–Eubacterium rectale* groups was observed after 4 weeks consumption of red wine polyphenols [130]. Mayta-Apaza et al. [129] have suggested that preexisting gut microbiota play a significant role in polyphenol-induced changes in the gut microbiota. Moreover, diet components possibly interfere with polyphenol–bacterial interaction. For example, the modulation of swine gut microbiota in the presence of purple sweet potato polyphenols was significantly affected depending upon the type of dietary fiber present in the mix [131]. Similarly, a change in the metabolism of rutin by fecal microorganisms was observed in the presence of fermentable fibers by Mansoorian et al. [132]. A gas chromatography–mass spectrometry analysis of human feces of four male and six female healthy participants revealed that the presence of highly fermented fibers such as ispaghula or pectin in the diet inhibits phenolic acid production from the catabolism of rutin by gut microbiota, whereas neither rutin nor quercetin had a detectable impact on short chain fatty acids production from the fermentation of these fibers. These findings suggest that a control fiber free diet is necessary to understand the polyphenols-–gut microbiota relationship during human studies, otherwise the presence of any type of dietary fiber would lead to misinterpretation of the polyphenol–gut association. Overall, with the studies in hand, the mechanism underlying the modulation of gut microbial species with different polyphenols is inconclusive, thus hindering the development of polyphenol-based treatments in psychotic disorders. However, the positive effect of polyphenols in managing antipsychotics-induced side effects and their potential to stabilize beneficial gut microorganisms is encouraging to consider their use alone or in combination with non-digestible fibers to elucidate their biotherapeutic potential (Figure 4c). Inclusively, more research and clinical trials are required to link the polyphenol and gut microbiota dysbiosis interplay with schizophrenia.

## 6. Conclusions and Future Perspectives

Current research studies provide an insight into how gut microbial diversity variation influences the neurotransmitters and metabolic pathways related to the central nervous system and contribute to the complex etiology of schizophrenia. It is vastly evident that the gut microbiota of schizophrenia patients is different than healthy individuals. The changes in the gut microbiota can be used as a biomarker for diagnostic purposes or as a treatment response if very specific microbial species are identified as schizophrenia signature microorganisms [12]. However, due to conflicting data, more dedicated research studies need to be conducted in this regard. Regulation of neurotransmitters is involved in neuron plasticity, memory, and learning processes. Recent findings have revealed the role of the gut microbiota in the regulation of different neurotransmitters to maintain brain hemostasis. Nonetheless, knowledge is still missing regarding the connotation of a particular gut microbial species and the dysfunction of neurotransmitters involved in this pathology. In fact, the aberrant function of a single neurotransmitter could not be associated to SCZ etiology. The dynamic alterations in gut microbial diversity and the lack of animal models makes it challenging to investigate the exact link of a particular microbial species and an individual neurotransmitter in health and disease. Understanding the etiology is pivotal to prevent the development of schizophrenia rather than treating the symptoms. Therefore, more complex experimental designs, the use of modern omics technologies, and analytical tools are recommended to unveil the exact mechanism that is linking gut microbial diversity and neurotransmitter regulation. Antipsychotics are the first medication typically prescribed to treat the symptoms of the disease. However, this treatment failed to cure all symptoms of schizophrenia and resulted in several side effects including reduced gut microbial diversity and increased metabolic dysfunction. Therefore, the use of nonpharmaceutical compounds can be considered to avoid antipsychotic-induced side effects and manipulate the symptoms of the disease.

Considering the modulation of the gut microbiome involved in the pathogenesis of the disease, manipulation of gut microbial diversity by phychobiotics can be a promising avenue for clinical research. There is substantial potential for the development of novel bioactive compounds that would modulate gut biodiversity and reduce the risk of schizophrenia. Nevertheless, detailed and large-scale studies of the relationship between schizophrenia, microbiota modulation by phychobiotics interventions, and the central nervous system are required to access the biotherapeutic potential of these compounds. New research studies must be designed to explore the possibility of whether probiotics, prebiotics, polyphenols alone or in combination may positively influence schizophrenia symptoms. These natural molecules may contribute to the treatment or management of the inflammatory process of the gastrointestinal system and gut–brain axis signaling pathways. Because the use of live bacteria (probiotics) has a transient effect, the fermentable fibrous compounds (prebiotics) and polyphenols can act as natural fertilizers for the maintenance of commensal healthy microbiota. However, to develop biotherapeutic-based treatment or prevention strategies for schizophrenia, an optimum dose of these compounds must be determined to avoid potential side-effects in humans with long-term exposure [124]. Moreover, a track record of dietary information of the participants, that is missing in current studies, would make future research efforts more conclusive. Overall, extensive and careful human trials are indispensable to confirm whether the modulation of the gut microbiota through pro/prebiotics and polyphenols could be a preventive or effective therapeutic approach for schizophrenia. Cautiously, consideration of the health, clinical condition, and preferences of patients and clinicians is recommended before choosing any treatment interventions for schizophrenic patients.

## Data Availability

Not applicable.
